# Benign fasciculation syndrome and migraine aura without headache as possible adverse events after BNT162b2 mRNA vaccination: a web-based survey

**DOI:** 10.3325/cmj.2023.64.430

**Published:** 2023-12

**Authors:** Grgur Salai, Đivo Ljubičić, Ruđer Novak, Lovorka Grgurević

**Affiliations:** 1 Department of Pulmonology, University Hospital Dubrava, Zagreb, Croatia; 2 Department of Internal Medicine, University of Zagreb, School of Medicine, Zagreb, Croatia; 3 Department of Proteomics, University of Zagreb, School of Medicine, Zagreb, Croatia; 4 Department of Anatomy Drago Perović, University of Zagreb School of Medicine, Zagreb, Croatia; 5 BIMIS – Biomedical Research Center Šalata, University of Zagreb School of Medicine, Zagreb, Croatia

## Abstract

**Aim:**

To determine the characteristics of patients who experienced muscle fasciculations and migraine auras without headache after BNT162b2 immunization.

**Methods:**

In January 2022, we published a case report that described a 48-year-old female patient who experienced muscle twitching and migraine auras without headache following BNT162b2 immunization. A self-administered online survey was sent to people who had written to us and complained of similar symptoms described in the case report (N = 20).

**Results:**

The survey was completed by 11 participants, of whom 10 reported muscle twitching following BNT162b2 immunization lasting a median of 14 (4-36.5) days. Five of these participants (50%) reported migraine auras without headache. Participants further reported on self-identified triggers that altered the intensity of their symptoms, such as anxiety or caffeine. Fifty percent of participants who got an acute SARS-CoV-2 infection (3/6) experienced increased muscle symptom intensity during the acute phase of the disease.

**Conclusion:**

To the best of our knowledge, our survey is the first to summarize patients' experiences of these phenomena occurring after BNT162b2 immunization. It is important to note that no causal relationship between vaccination and these phenomena can be inferred.

Vaccination against SARS-CoV-2 has decreased COVID-19 mortality and severe disease forms (1). Several neurological adverse events following immunization (AEFI) were reported after the BNT162b2 vaccination, but no causality could be inferred (2,3).

Benign muscle fasciculations are visible, spontaneous muscle fiber contractions that occur intermittently (3-5). Migraine aura without headache is a recurrent disorder manifesting in attacks of reversible focal neurological symptoms, neither accompanied nor followed by headache (6,7).

In January 2022, we published a case report regarding muscle twitching and migraine aura without headache in a 48-year-old woman as potential AEFI following BNT162b2 immunization. Muscle fasciculations started to intermittently appear six days after immunization. Furthermore, she experienced migraine auras with visual kaleidoscope-like phenomena on two occasions (3). Muscle twitching and migraine-related phenomena following COVID-vaccines have seldom been reported in the literature, but have been reported by laypeople on social networks (8-13).

Following the publication of our case report, we received several e-mails from people who complained of similar ailments. In this report, we aimed to summarize their experience by administering them a survey.

## Patients and methods

**Figure 1:**
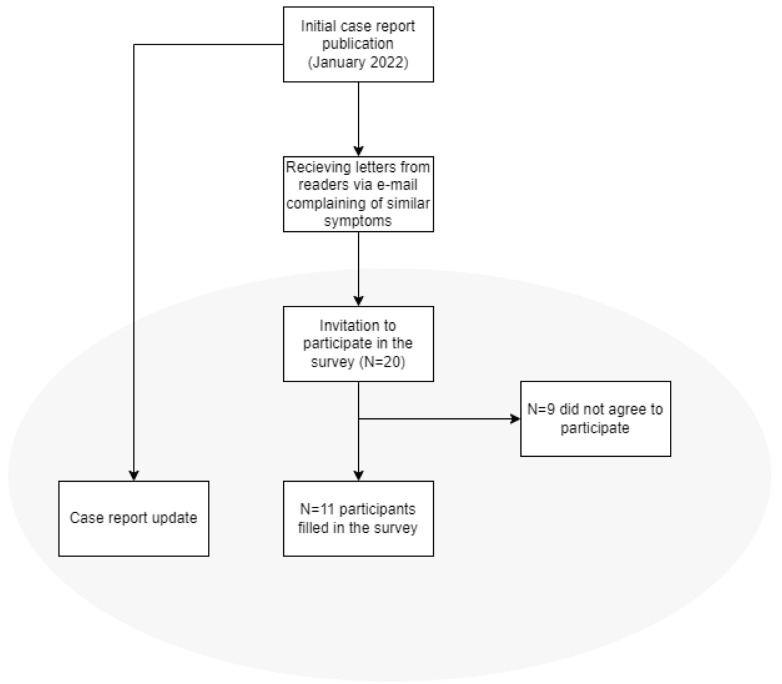
Flowchart of the study.

This study was designed as an online self-administered survey and was approved by the Institutional Ethics Committee of the School of Medicine, University of Zagreb (380-59-10106-22-111/79). Non-probability sampling was used to select participants: people that reached out to the authors of Salai et al from 2022 (3) were sent an email in which they were asked to participate in a self-administered survey. The study design is depicted in Figure 1.

The survey was written in English (Supplementary Material 1) and consisted of questions regarding demographic information, twitching and/or aura symptoms, vaccination/infection status, and the EuroQoL (EQ-5D-5L) questionnaire. EQ-5D-5L was employed to quantify the participants’ self-reported health using a standardized testing tool (14,15).

Qualitative variables are presented as numbers and percentages. Data normality was tested with the Kolmogorov-Smirnov test. Parametric variables are presented as mean ± standard deviation (SD); non-parametric variables are presented as median (Q1-Q3). Friedman's test was employed to assess the difference between two time points. Data analysis was conducted with MedCalc® Statistical Software, version 20.106 (16), and JAMOVI 2020 (17).

## Results

### Case report update

After the publication of the case report (3), our patient experienced a gradual decrease in symptom intensity. In June 2022, she experienced a SARS-CoV-2 infection with mild respiratory symptoms, during which she developed worsening muscle fasciculations and had another brief episode of migraine aura. Furthermore, during the post-acute period, her fasciculations were increased for two weeks, after which the symptoms gradually decreased. Currently, she is asymptomatic.

### Survey analysis

The initial invitations to participate in the survey were sent to 20 email addresses; 11 (55%) people agreed to participate (4 women). Participants' characteristics are shown in Table 1. No participant had a history of neurological illness; one had previously experienced muscle twitching, but this happened sporadically.

**Table 1:** Participants' demographic characteristics

Participants' characteristics	N (%)
**Sex**	
male	7 (63.6)
female	4 (36.4)
**Age (median)**	32 (24.5-46)
**English-speaking country**	
total	10 (90.9)
Australia	3
Canada	2
United Kingdom	2
United States of America	3
**Non-English-speaking country**	
Austria	1 (9.1)
**Healthcare workers**	
total	3 (27.3)
physician	1
non-physician	2
**Level of education**	
high-school	2 (18.1)
bachelor's degree	5 (45.5)
master's degree	3 (27.3)
PhD	1 (9.1)
**Pre-vaccination medical history**	
**Previous psychiatric illnesses**	
anxiety	1
attention deficit hyperactivity disorder	1
none	9
**Previous respiratory illnesses**	
asthma	1
obstructive sleep apnea	1
none	9

All participants received the BNT162b2 vaccine. The median number of doses was 2 (1-2). One participant did not ascribe muscle twitching to vaccination but to the post-acute period of SARS-CoV-2 infection. Ten participants ascribed their symptoms to immunization. Eight participants complained of AEFI after the first, and two after the second vaccination dose. In the post-vaccination phase, 10 participants experienced muscle twitching. The median time from vaccination to fasciculation onset was 14 (4-36.5) days. Five (50%) participants experienced migraine auras without headaches. All participants sought medical help for these phenomena.

**Figure 2:**
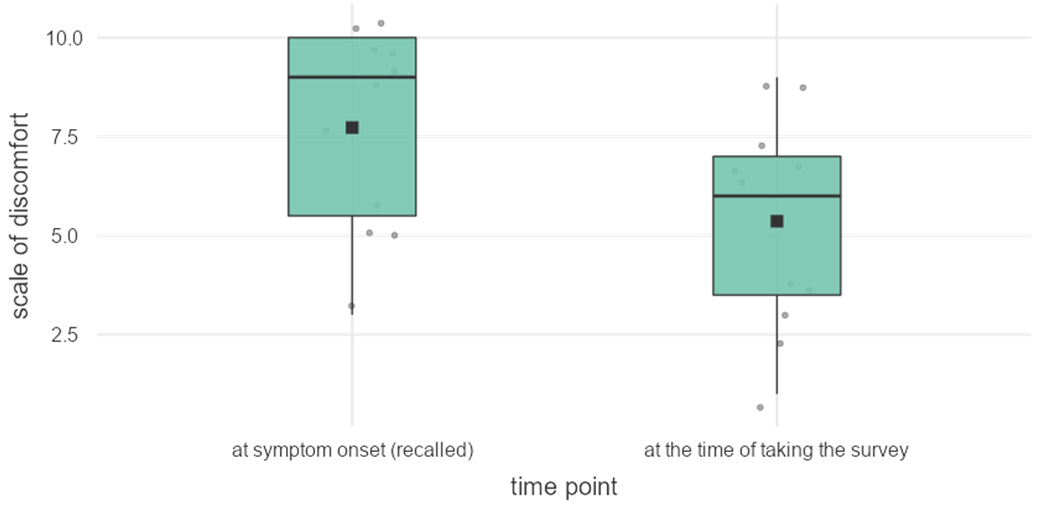
Self-assessment of overall discomfort at symptom onset (recalled) and at the time of taking the survey on a scale from 0 (no discomfort) to 10 (extreme discomfort). Black squares indicate arithmetical means.

The survey was filled out 321 (270-405) days after the symptom onset. Five participants stated that symptom intensity was reduced over time, 5 stated that the intensity remained the same, and 1 stated that symptoms increased in intensity. When asked to recall the level of discomfort at the time of symptom onset on a scale from 0 (no discomfort) to 10 (extreme discomfort), the median result was 9 (7-10) vs 6 (3.5-7) at the time of taking the survey (χ^2^ = 1.6, *P* = 0.206) (Figure 2).

When asked what factors led toward the decrease in symptom intensity, they identified the following: magnesium supplements (n = 3), physical movement (n = 1), acetazolamide (dosage unknown) (n = 1), and “vitamin supplements” (n = 1). The factors that aggravated their symptoms were caffeine (n = 2), physical exercise (n = 2), and anxiety and emotional stress (n = 2).

Six participants had a SARS-CoV-2 infection, of whom 3 experienced muscle twitching during acute infection. Participants tested positive after a median of 270 (205-294) days after receiving the dose after which they noticed AEFIs. None experienced migraine auras during acute infection. Nine participants filled in the EuroQoL-5D-5L portion of the survey (Table 2). Problems across five standardized dimensions were either slight or moderate. All participants reported problems in the “pain/discomfort” dimension. When asked how good or bad their health was today, the median response was 75 (50-80) out of maximum 100.

**Table 2:** EuroQoL-5D-5L questionnaire results, reported as the number of participants per dimension per level and as percentage (based on a number of participants who completed the questionnaire)

	Mobility N (%)	Self-care N (%)	Usual activities N (%)	Pain/discomfort N (%)	Anxiety/depression N (%)
**Level 1** No problems	6 (66.6)	9 (100)	3 (33.3)	0	4 (44.4)
**Level 2** Slight problems	1 (11.1)	0	5 (55.5)	3 (33.3)	2 (22.2)
**Level 3** Moderate problems	2 (22.2)	0	1 (11.1)	6 (66.6)	3 (33.3)
**Level 4** Severe problems	0	0	0	0	0
**Level 5** Unable to do	0	0	0	0	0
**Complete data**	9	9	9	9	9
**Missing data**	2	2	2	2	2
**Total**	11	11	11	11	11

## Discussion

We conducted a non-probability sampling-based online survey aiming to further explore muscle fasciculations and migraine auras as potential AEFI. Our survey indicated a non-significant decrease in self-assessed discomfort level at the time of taking the survey compared with the recalled discomfort level at the time of symptom onset. A half of the participants who had SARS-CoV-2 infection after vaccination experienced increased muscle twitching, and the same was true for the patient we presented in our original case report. Muscle fasciculations have previously been described to occur during acute COVID-19 (18). Furthermore, in a telephone interview-based study of patients with previously diagnosed benign fasciculation syndrome (BFS), a subset of patients self-reported exacerbation of symptoms during acute viral infections (19). It has been hypothesized that viral infections might impact the course of BFS by triggering autoimmunity (20-22). Muscle spasms after vaccination are generally considered to be mild and self-limiting AEFI (23,24). While BFS may be uncomfortable and present a high psychological burden, its disease course is benign (25).

Our study has several major limitations. First, it was based on a self-administered online survey and is prone to self-reporting bias (26); additionally, no objective clinical measurements were employed. Second, we used non-probability sampling, enrolling people who sent us an e-mail, usually because they found similarity between the symptoms we previously described (3) and the phenomena they experienced, which makes this study additionally prone to sampling bias (27). Besides this, our previous case report was in English, so the accessibility to it, as well as the ability to contact the authors, was limited to English speakers. Furthermore, the study had a small sample size and lacked a control group (which is inherent to our study design). An important point to consider is also the bidirectional relationship between health anxiety and BFS (5,28), as it is possible that searching for information of muscle fasciculation could exacerbate the symptoms. Finally, the potential impact of SARS-CoV-2 infection on muscle fasciculation in our study participants might be a source of confounding when assessing symptom severity.

To conclude, we reported a summarized experience of a small subset of people who had read our previous report and decided to contact us (most likely) due to the similarity with their own condition. It is very important to highlight that no causal inference can be made regarding BNT162b2 vaccination and muscle fasciculations or migraine auras without headache. The primary intention of our study was to report the presence of individuals who had experienced similar phenomena as our patient. To emphasize, muscle twitching and/or migraine auras without headaches that occur following SARS-CoV-2 vaccination seem to be extremely rare events: clinical studies with systematical and objective measurements are required in order to elucidate the potential relationship between BNT162b2 vaccination and these phenomena in certain individuals.

## References

[1] Huang Y-Z Kuan C-C Vaccination to reduce severe COVID-19 and mortality in COVID-19 patients: a systematic review and meta-analysis. Eur Rev Med Pharmacol Sci 2022 26 1770 6 35302230 10.26355/eurrev_202203_28248

[2] Khayat-Khoei M Bhattacharyya S Katz J Harrison D Tauhid S Bruso P COVID-19 mRNA vaccination leading to CNS inflammation: a case series. J Neurol 2022 269 1093 106 10.1007/s00415-021-10780-7 34480607 PMC8417681

[3] Salai G Bilic E Primorac D Lakusic DM Bilic H Lazibat I Benign fasciculation syndrome and migraine aura without headache: possible rare side effects of the BNT162b2 mRNA baccine? A Case Report and a Potential Hypothesis. Vaccines (Basel) 2022 10 117 10.3390/vaccines10010117 35062778 PMC8780563

[4] Leite MAA Orsini M De Freitas MRG Pereira JS Gobbi FHP Bastos VH Another perspective on fasciculations: when is it not caused by the classic form of amyotrophic lateral sclerosis or progressive spinal atrophy? Neurol Int 2014 6 10.4081/ni.2014.5208 25309711 PMC4192433

[5] Filippakis A Jara J Ventura N Scala S Scopa C Ruthazer R A prospective study of benign fasciculation syndrome and anxiety. Muscle Nerve 2018 58 852 4 10.1002/mus.26193 30028521

[6] Shah DR Dilwali S Friedman DI Current aura without headache. Curr Pain Headache Rep 2018 22 77 10.1007/s11916-018-0725-1 30225597

[7] The International Classification of Headache Disorders3rd edition (beta version)Cephalalgia 2013 33 629 808 10.1177/0333102413485658 23771276

[8] Yılmaz BÖ Benign Fasciculation Syndrome Developing after COVID Vaccine (Sinovac/CoronaVac). Case Reports Clin Med. 2022 11 218 20 10.4236/crcm.2022.116032

[9] Consoli S Dono F Evangelista G D’Apolito M Travaglini D Onofrj M Status migrainosus: a potential adverse reaction to Comirnaty (BNT162b2, BioNtech/Pfizer) COVID-19 vaccine—a case report. Neurol Sci 2021 34807361 10.1007/s10072-021-05741-x PMC8607053

[10] Waliszewska-Prosół M Budrewicz S The unusual course of a migraine attack during COVID-19 infection — Case studies of three patients. J Infect Public Health 2021 14 903 5 10.1016/j.jiph.2021.04.013 34119843 PMC8101002

[11] Rattanawong W Akaratanawat W Tepmongkol S Chutinet A Tantivatana J Suwanwela NC Acute prolonged motor aura resembling ischemic stroke after COVID − 19 vaccination (CoronaVac): the first case report. J Headache Pain 2021 22 93 10.1186/s10194-021-01311-w 34384351 PMC8358547

[12] Quora.com. Has anyone experienced muscle twitching and crippling muscle weakness a few weeks after receiving the Pfizer vaccine? Available from: https://www.quora.com/Has-anyone-experienced-muscle-twitching-and-crippling-muscle-weakness-a-few-weeks-after-receiving-the-Pfizer-vaccine*.* Accessed: October 10, 2023.

[13] Reddit.com. Twitching after covid vaccine. Available from: https://www.reddit.com/r/BFS/comments/r7b86b/twitching_after_covid_vaccine_did_yours_stopped/*.* Accessed: October 10, 2023.

[14] Rabin R de Charro F EQ-5D: a measure of health status from the EuroQol Group. Ann Med 2001 33 337 43 10.3109/07853890109002087 11491192

[15] Feng Y-S Kohlmann T Janssen MF Buchholz I Psychometric properties of the EQ-5D-5L: a systematic review of the literature Qual Life Res 2021 30 647 73 10.1007/s11136-020-02688-y 33284428 PMC7952346

[16] MedCalc® Statistical Software. Ostend, Belgium: MedCalc Software Ltd; 2022. Available from: https://www.medcalc.org*.* Accessed: December 20, 2023.

[17] The-jamovi-project. JAMOVI. 2020. Available from: https://www.jamovi.org. Accessed: December 20, 2023.

[18] Paliwal VK Garg RK Gupta A Tejan N Neuromuscular presentations in patients with COVID-19. Neurol Sci 2020 41 3039 56 10.1007/s10072-020-04708-8 32935156 PMC7491599

[19] Blexrud MD Windebank AJ Daube JR Long-term follow-up of 121 patients with benign fasciculations. Ann Neurol 1993 34 622 5 10.1002/ana.410340419 8215252

[20] Kolberg ES A viral origin for benign fasciculation syndrome? Neurol Sci 2021 42 1621 2 10.1007/s10072-020-04888-3 33169194

[21] Imam I Edwards S Hanemann CO Acquired neuromyotonia following upper respiratory tract infection: a case report. Cases J 2009 2 7952 10.4076/1757-1626-2-7952 19918441 PMC2769391

[22] Vernino S Lennon VA Ion channel and striational antibodies define a continuum of autoimmune neuromuscular hyperexcitability. Muscle Nerve 2002 26 702 7 10.1002/mus.10266 12402293

[23] Hosseini R Askari N A review of neurological side effects of COVID-19 vaccination. Eur J Med Res 2023 28 102 10.1186/s40001-023-00992-0 36841774 PMC9959958

[24] Assiri SA Althaqafi RMM Alswat K Alghamdi AA Alomairi NE Nemenqani DM Post COVID-19 vaccination-associated neurological complications. Neuropsychiatr Dis Treat 2022 18 137 54 10.2147/NDT.S343438 35140464 PMC8818972

[25] Filippakis A Jara J Ventura N Ruthazer R Russell J Ho D A prospective study of benign fasciculation syndrome (S45.007). Neurology 2017 88 16 Supplement S45.007 10.1212/WNL.88.16_supplement.S45.007 30028521

[26] Bauhoff S. Self-Report bias in estimating cross-sectional and treatment effects bt - encyclopedia of quality of life and well-being research. In: Michalos AC, editor. Encyclopedia of Quality of life and well-being research. Dordrecht: Springer Netherlands; 2014. p. 5798-800.

[27] Tyrer S Heyman B Sampling in epidemiological research: issues, hazards and pitfalls. BJPsych Bull 2016 40 57 60 10.1192/pb.bp.114.050203 27087985 PMC4817645

[28] Blackman G Cherfi Y Morrin H Ellis CM Bashford J Ruths F The association between benign fasciculations and health anxiety: a report of two cases and a systematic review of the literature. Psychosomatics 2019 60 499 507 10.1016/j.psym.2019.04.001 31174866

